# Quantitative assessment of relative peripheral refraction in children with different refractive statuses and its associations with ocular biometry

**DOI:** 10.3389/fmed.2026.1711559

**Published:** 2026-02-02

**Authors:** Chaoying Ye, Xingxue Zhu, Yangfan Xu, Yuliang Wang, Yujia Liu, Jianmin Shang, Xiaomei Qu

**Affiliations:** 1 Department of Ophthalmology and Vision Science, Eye & ENT Hospital, Fudan University, Shanghai, China; 2 NHC Key Laboratory of Myopia, Fudan University, Shanghai, China; 3 Laboratory of Myopia, Chinese Academy of Medical Sciences, Shanghai, China

**Keywords:** multispectral refraction topography, ocular biometry, refractive error, relative peripheral refraction, retinal defocus

## Abstract

**Background:**

This study aimed to evaluate the distribution of relative peripheral refraction (RPR) and its relationship with ocular biometry in Chinese children of different refractive statuses.

**Methods:**

This study included 309 participants aged 4 to 14 years who were divided into three groups based on refraction: hyperopia, emmetropia, and myopia. IOLMaster 700 was used to acquire ocular biometry data, and RPR was measured using multispectral refraction topography. Refraction difference values (RDVs) were used to describe the RPR and included the total defocus (TRDV), defocus at 0° to 15° (RDV15), 15° to 30° (RDV15–30), 30° to 45° (RDV30–45), and 45° to 53° (RDV45–53) eccentricities, and superior (RDV-S), inferior (RDV-I), temporal (RDV-T), and nasal (RDV-N) quadrants.

**Results:**

In participants with emmetropia and myopia, RPR values became more positive as the distance from the foveal pit increased, whereas hyperopic participants showed a decrease in relative hyperopic defocus in the RDV45–53. Beyond 30° eccentricity, there were significant differences between the refractive groups; those with emmetropia and myopia had significantly higher RPR values than those with hyperopia. The spherical equivalent was negatively correlated with RDV-T (*β* = −0.31, *p* < 0.01) and TRDV (*β* = −0.26, *p* < 0.01). In myopic children, the correlation extended to multiple peripheral regions with increasing degrees of myopia. The ocular parameter most strongly associated with RDV45–53 was the axial length/corneal radius of curvature (AL/R, *β* = 0.48, *p* < 0.01).

**Conclusion:**

In emmetropic and myopic children, RPR values became progressively positive with greater eccentricity, indicating a relative peripheral hyperopic shift. In contrast, hyperopic children showed a reduction in the RPR beyond 30° eccentricity. The spherical equivalent was negatively correlated with temporal and total RPR, and AL/R showed the strongest association with far peripheral RPR.

## 1 Introduction

The prevalence of myopia is increasing worldwide and has been reported to affect more than 60% of children aged less than 12 years, up to 80% of children aged less than 16 years, and more than 90% of college students in China (1, 2). Myopia can also cause numerous inconveniences in daily life and result in pathological conditions such as retinal detachment, cataracts, and myopic macular degeneration (3, 4). Therefore, the prevention and control of myopia are crucial.

The relationship between relative peripheral refraction (RPR) and myopia has been extensively investigated. Although Hoogerheide et al. (5) first documented relative hyperopic defocus in the peripheral retina of established myopic eyes, contemporary studies have confirmed that this pattern emerges as a result of myopia (6). Rotolo et al. (7) confirmed that relative peripheral refraction cannot predict myopia onset or progression in Mediterranean children, and interventions modifying peripheral refraction likely act through mechanisms beyond RPR. Atchison and Rosén (8) established peripheral hyperopia as an anatomical characteristic of myopic eyes post-onset. These results indicate that RPR is more likely to be a concurrent phenomenon than an initiating factor for myopia.

Multispectral refraction topography (MRT) is a newly developed imaging technique that can quantify retinal refraction across a 53° posterior pole field. It uses multispectral illumination to acquire fundus images, and then, proprietary algorithms computationally separate the refractive data from them at each retinal position, resulting in the generation of high-resolution dioptric topographic maps. The reliability and repeatability of this technique have been verified in previous studies (9, 10). Compared with the current gold standard, the open-field autorefractor (WAM-5500; Grand Seiko Co., Ltd., Hiroshima, Japan), MRT is easier to operate and more time-efficient (11–13). MRT also offers a wider measurement range than the Hartmann–Shack wavefront sensor technique (Wavefront Sciences Inc., Albuquerque, NM, USA) (14, 15). These capabilities position MRT as a suitable tool for comparing RPR profiles.

Recent studies have described RPR in children with different refractive statuses (16–22). However, the application of MRT to compare the RPR profiles among children with different refractive statuses remains unexplored. A better understanding of the relationship between peripheral defocus and ocular parameters may contribute to improved strategies for the prevention and control of myopia.

## 2 Methods

### 2.1 Participants and enrollment

Participants were consecutively recruited from patients who visited the Eye & ENT Hospital of Fudan University (Shanghai, China) with the following inclusion criteria: (1) age 4 to 14 years; (2) cycloplegic spherical equivalent (SE) between −9.00 and +10.00 D and astigmatism ≤ − 1.50 D; and (3) best-corrected distance visual acuity of 0.10 logMAR or better. The exclusion criteria were as follows: (1) cornea that was too flat (K < 42.00 D) or too steep (K > 47.00 D); (2) active eye disease or a history of organic eye diseases, such as cataracts, glaucoma, or conical cornea; (3) previous or current myopia control treatment, such as atropine, pirenzepine drops, orthokeratology lenses, or soft multifocal contact lenses; and (4) major systemic disease.

This cross-sectional study was approved by the Ethics Committee of the Eye & ENT Hospital of Fudan University and conducted in accordance with the tenets of the Declaration of Helsinki. Written informed consent was obtained from each participant.

### 2.2 Biometric measurements

The IOLMaster 700 (Carl Zeiss Meditec, Inc., Dublin, CA, USA) was used to measure the central corneal thickness (CCT), anterior chamber depth (ACD), aqueous depth (AQD), lens thickness (LT), and axial length (AL) five times in succession. The corneal curvature was also assessed, resulting in flat (K1) and steep (K2) curvatures. The mean curvature power (Km) represents the average of the two. The mean corneal radius of curvature (R) is derived from Km.

The commonly used agents for cycloplegia are 0.5% tropicamide eye drops. Five consecutive eye drops were administered at 5-min intervals, followed by assessment 30 min later to measure the SE. Best-corrected visual acuity (BCVA) was measured at a distance of 4 m and converted to the logarithm of the minimum angle of resolution (logMAR). Participants were categorized into three groups according to the SE determined by subjective refraction: hyperopia was defined as SE > +0.75 D, emmetropia was defined as −0.50 D < SE ≤ +0.75 D, and myopia was defined as SE ≤ −0.50 D. The myopia group was further subdivided into low myopia (−3.00 D < SE ≤ −0.50 D) and moderate-to-high myopia (SE ≤ −3.00 D).

Relative peripheral refraction was acquired using MRT (MSIC2008, ShengDa TongZe, ShenZhen, China) under cycloplegia for RDVs, which measures the difference between peripheral retinal refraction and central foveal, with positive values representing relative hyperopic defocus and negative values representing relative myopic defocus. The RDVs included RDV15, RDV15–30, RDV30–45, and RDV45–53 within the 15°, 15°–30°, 30°–45°, and 45°–53° ranges, respectively, centered on the foveal pit. Total defocus (TRDV within the 53° range) was divided into four quadrants: superior defocus (RDV-S), inferior defocus (RDV-I), temporal defocus (RDV-T), and nasal defocus (RDV-N). One examiner averaged the measurements over three replicates, and only results with a quality score of >80% were recorded for further analysis.

### 2.3 Statistical analysis

The right eye of each participant was analyzed. The Kolmogorov–Smirnov test was used to determine the normality of continuous variables, and Bartlett’s test was used for homogeneity of variance. For normally distributed variables with homogeneous variance, expressed as mean ± standard deviation, a one-way analysis of variance or analysis of covariance was performed, followed by the least significant difference test if significant differences existed. Variables that were non-normally distributed and expressed as medians (Q1 and Q3) were examined using the Kruskal–Wallis non-parametric test, and significant differences were compared pairwise using Bonferroni correction. Categorical variables were expressed as frequencies and percentages and analyzed using the chi-squared test. Spearman’s correlation, partial correlation, multivariate linear regression analyses, and a generalized linear model were used to explore correlations between the parameters. All analyses were performed using SPSS v20.0, and a two-tailed *p-*value of < 0.05 was considered statistically significant. Graphs were created using GraphPad Prism, version 9.5.0.

## 3 Results

A total of 309 children and adolescents, aged 4 to 14 years, with SE ranging from −8.625 D to +9.625 D and AL from 20.10 mm to 26.97 mm, were enrolled. The distribution characteristics of participants with different refractive statuses are presented in Table 1.

**Table 1:** Demographic information and baseline characteristics of the participants.

Parameters	Total	Hyperopia	Emmetropia	Myopia				*P*_Total_
				Total	Low myopia	Moderate-to-high myopia	*P*_M_	
Number (%)	309 (100.00)	92 (29.77)	52 (16.83)	165 (53.40)	118 (38.19)	47 (15.21)	–	–
Sex (%)								
Female	159 (51.46)	45 (48.91)	24 (46.15)	90 (54.55)	73 (61.86)	17 (36.17)	<0.01^†,^ **	0.48^†^
Male	150 (48.54)	47 (51.19)	28 (53.85)	75 (45.45)	45 (38.14)	30 (63.83)		
Age (years)	8.26 ± 2.11	7.30 ± 2.36	8.21 ± 1.76	8.80 ± 1.88	8.47 ± 1.71	9.64 ± 2.03	<0.01**	<0.01**
SE (D)	−0.32 ± 2.88	3.11 ± 1.98	0.13 ± 0.38	−2.36 ± 1.59	−1.54 ± 0.65	−4.43 ± 1.36	<0.01**	<0.01**
AL (mm)	23.64 ± 1.34	22.19 ± 0.98	23.60 ± 0.73	24.45 ± 0.92	24.17 ± 0.78	25.17 ± 0.87	<0.01^‡,^**	<0.01^‡,^**

**p < 0.01; *P*_M_, the *p*-value for the comparison between low and moderate-to-high myopia subgroups; *P*_Total_, the *p*-value for the comparisons across all three refractive groups; SE, spherical equivalent; AL, axial length.

^†^Chi-squared test was performed.

^‡^A one-way analysis of variance was performed; the remaining parameters were analyzed using the Kruskal–Wallis non-parametric test.

**Figure 1:**
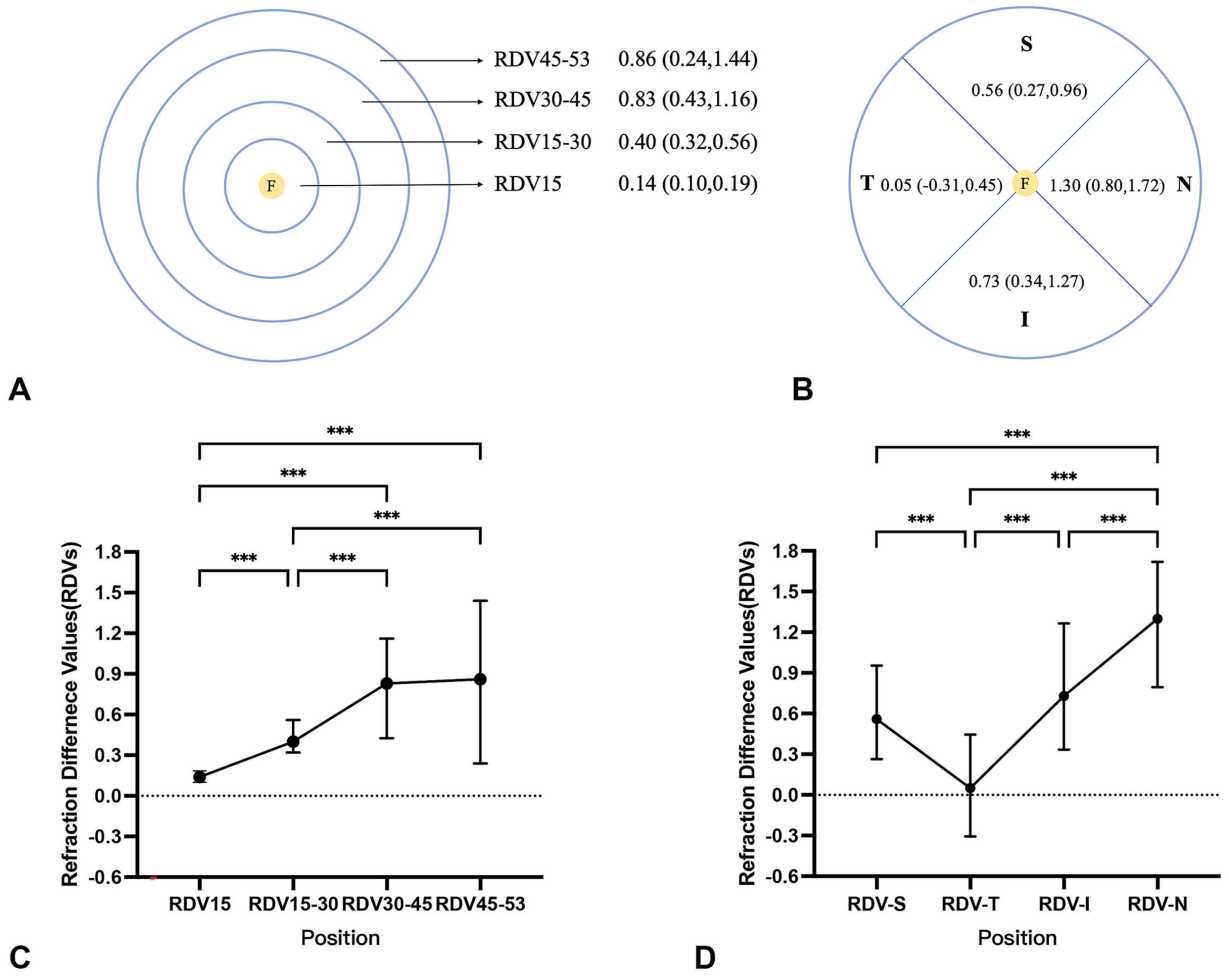
Comparison of RDVs at different eccentricities and in different quadrants for all participants. ***p < 0.001; RDV, refraction difference values. RDVs are expressed as medians (Q1, Q3) at different eccentricities **(A)** and quadrants **(B)**, analyzed using the Kruskal–Wallis non-parametric test, and Bonferroni correction was used to adjust the significance values for different eccentricities **(C)** and quadrants **(D)**. The following ranges were centered on the foveal pit: RDV15, within a 15° range; RDV15–30, within a 15°–30° range; RDV30–45, within a 30°–45° range; and RDV45–53, within a 45°–53° range. RDV-S, superior defocus; RDV-I, inferior defocus; RDV-T, temporal defocus; RDV-N, nasal defocus.

For all children, at different eccentricities, as shown in Figure 1, the RDVs exhibited positive values, indicating relative hyperopic defocus, and progressively increased with distance from the foveal pit. Relative peripheral refraction exhibited asymmetry across the quadrants, with RDV-N being the most relatively hyperopic and RDV-T the least.

**Figure 2:**
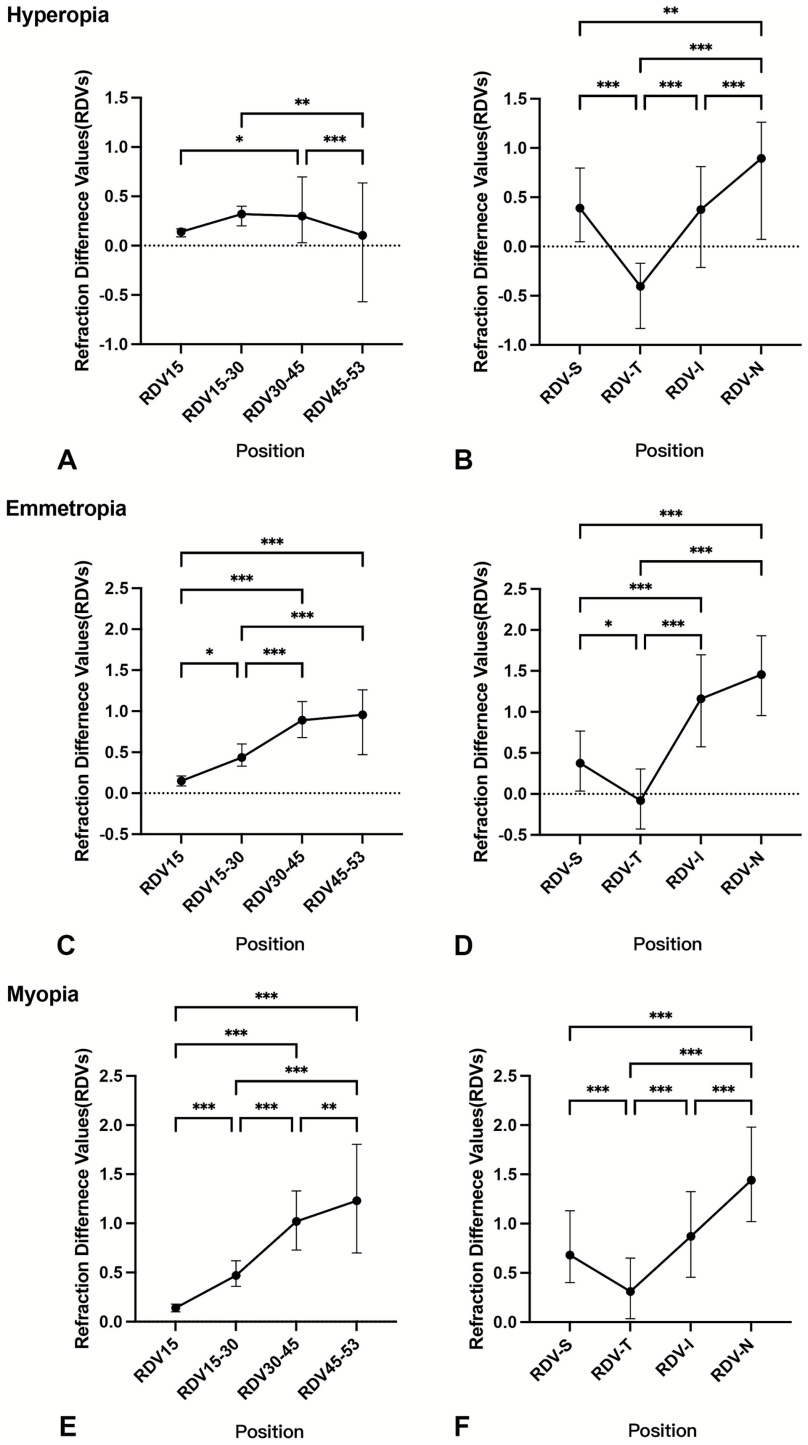
Comparison of RDVs at different eccentricities and in different quadrants by refractive status. *p < 0.05 **p < 0.01 ***p < 0.001; RDV, refraction difference values. The RDVs were analyzed using the Kruskal–Wallis non-parametric test, with Bonferroni correction applied to adjust the significance values for the hyperopia group **(A,B)**, emmetropia group **(C,D)**, and myopia group **(E,F)** at different eccentricities and quadrants.

Children with emmetropia or myopia exhibited a gradual increase in relative hyperopic defocus as distance from the foveal pit increased. However, among children with hyperopia, there was a slight decrease in hyperopic defocus in the RDV45–53 (Figure 2). The trends of the RDVs in the four quadrants across the different refractive statuses were similar: RDV-N > RDV-I > RDV-S > RDV-T.

**Figure 3:**
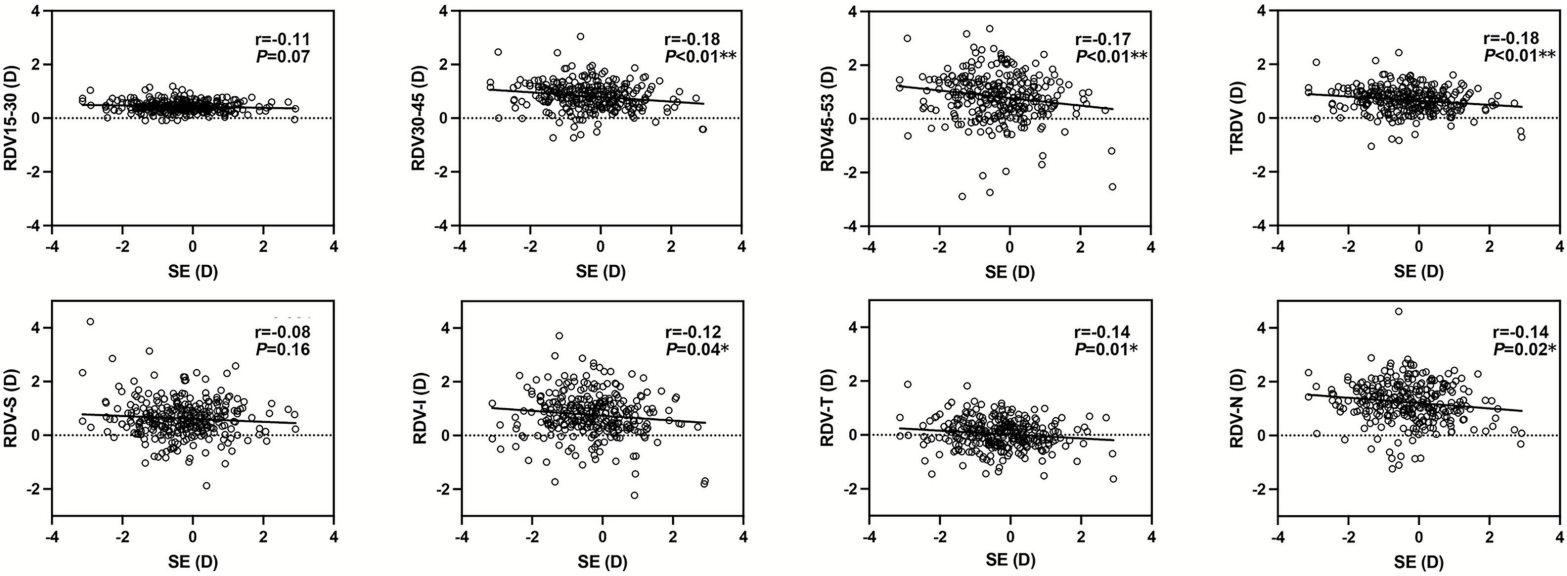
Partial correlation between refraction difference values (RDVs) and spherical equivalent (SE). ^*^p < 0.05 **p < 0.01; *r*, partial correlation coefficient. The controlling factors were the axial length/radius of curvature (AL/R) and age. The following ranges were centered on the foveal pit: RDV15−30, within a 15°–30° range; RDV30–45, within a 30°–45° range; and RDV45–53, within a 45°–53° range. TRDV, within the total measuring range of a 53° circle. RDV-S, superior defocus; RDV-I, inferior defocus; RDV-T, temporal defocus; RDV-N, nasal defocus.

After controlling for AL/R and age, children with hyperopia exhibited less relative hyperopic defocus beyond 30° retinal eccentricity than children with emmetropia or myopia (RDV30–45: *p* = 0.01, RDV45–53: *p* < 0.01), as shown in Table 2. However, the difference in defocus between children with emmetropia and myopia was not significant, with the exception of RDV-T (*p* < 0.05). Partial correlation analyses also revealed a significant negative correlation between the SE and RDVs beyond 30° eccentricity (Figure 3). Incorporating these significant variables into a stepwise multiple linear regression model revealed that SE was primarily correlated with TRDV (*β* = − 0.26, *t* = − 3.80, *p* < 0.01), RDV-T (*β* = − 0.31, *t* = − 4.69, *p* < 0.01), and age (*β* = − 0.20, *t* = − 4.19, *p* < 0.01).

**Table 2:** Comparison of refraction difference values at different eccentricities and quadrants of the eye by refractive status after controlling for AL/R and age.

Eccentricity median (Q1, Q3)	Total *n* = 309	Hyperopia *n* = 92	Emmetropia *n* = 52	Myopia *n* = 165	*p*-value
RDV15	0.14 (0.10, 0.19)	0.14 (0.09, 0.17)	0.15 (0.09, 0.21)	0.14 (0.10, 0.18)	0.30
RDV15–30	0.40 (0.32, 0.56)	0.32 (0.20, 0.40)	0.44 (0.33, 0.60)	0.47 (0.36, 0.62)	0.12
RDV30–45^†^	0.82 ± 0.60	0.36 ± 0.52	0.90 ± 0.46^a^	1.05 ± 0.53^c^	0.01^‡,^*
RDV45-53	0.86 (0.24, 1.44)	0.11 (−0.57, 0.64)	0.96 (0.47, 1.26)^a^	1.23 (0.70, 1.81)^c^	0.002**
TRDV^†^	0.67 ± 0.55	0.23 ± 0.48	0.74 ± 0.43^a^	0.89 ± 0.47^c^	<0.001^‡,^**
RDV-S	0.56 (0.27, 0.96)	0.39 (0.05, 0.80)	0.38 (0.04, 0.77)	0.68 (0.40, 1.13)	0.085
RDV-T	0.05 (−0.31, 0.45)	−0.41 (−0.83, –0.17)	−0.08 (−0.43, 0.31)^a^	0.31 (0.04, 0.65)^b, c^	<0.001**
RDV-I	0.73 (0.34, 1.27)	0.38 (−0.21, 0.81)	1.16 (0.58, 1.70)^a^	0.87 (0.46, 1.33)	<0.001**
RDV-N	1.30 (0.80, 1.72)	0.90 (0.07, 1.26)	1.46 (0.96, 1.93)^a^	1.44 (1.02, 1.98)	0.02

*p < 0.05 **p < 0.01; *n*, number; AL/R, axial length/radius of curvature; RDV, refraction difference values.

^†^Data with normal distribution are expressed as mean ± standard deviation.

^‡^Analysis of covariance (ANCOVA) and least significant difference post-hoc testing were performed; the remaining variables were analyzed using a generalized linear model (GLM).

^a^Significant difference between hyperopia and emmetropia (*p* < 0.05).

^b^Significant difference between emmetropia and myopia (*p* < 0.05).

^c^Significant difference between hyperopia and myopia (*p* < 0.05).

The following ranges were centered on the foveal pit: RDV15, within a 15° range; RDV15–30, within a 15° − 30° range; RDV30–45, within a 30° − 45° range; and RDV45–53, within a 45°–53° range. TRDV, within the total measuring range of a 53° circle. RDV-S, superior defocus; RDV-I, inferior defocus; RDV-T, temporal defocus; and RDV-N, nasal defocus.

To further explore the peripheral defocus across myopia severity, an additional subgroup analysis was performed (Supplementary Table 1). After adjusting for AL/R, age, and sex (Supplementary Table 2), the SE exhibited significant correlations between RDV15–30 (*r* = −0.23, *p* < 0.01) and RDV-S (*r* = −0.25, *p* < 0.01). Within the low myopia group, SE showed a significant negative correlation only with RDV-N (*r* = 0.24, *p* < 0.01), while in the moderate-to-high myopia group, SE was negatively correlated with RDVs beyond 15° eccentricity and TRDV (*r* = −0.36, *p* = 0.02), as well as with RDV-S (*r* = −0.53, *p* < 0.01) and RDV-N (*r* = −0.32, *p* = 0.04).

**Figure 4:**
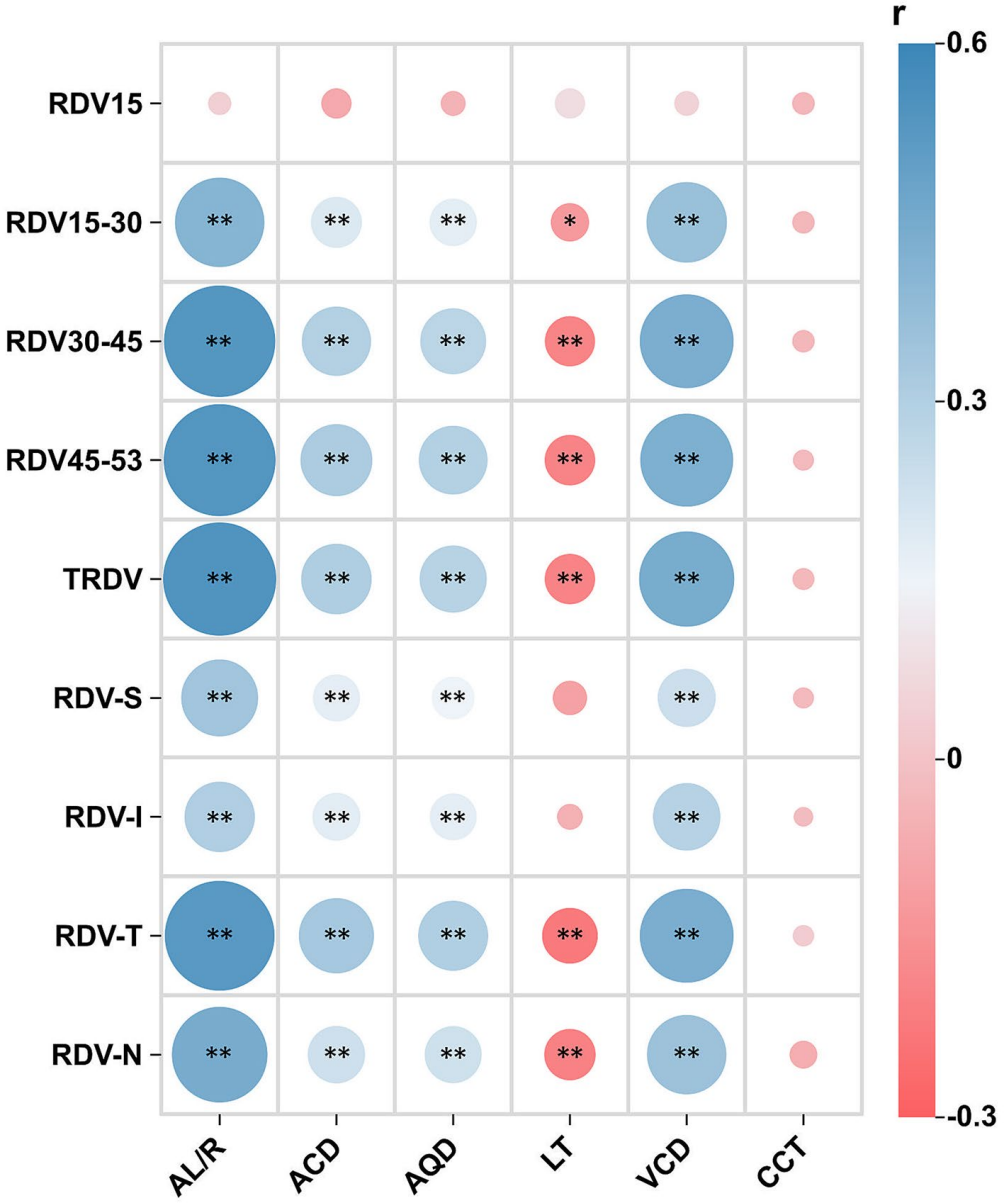
Correlation between refraction difference values (RDVs) and ocular biometry. *p < 0.05 **p < 0.01; *r*, correlation coefficient. The following ranges were centered on the foveal pit: RDV15, within a 15° range; RDV15–30, within a 15° − 30° range; RDV30–45, within a 30° − 45° range; and RDV45–53, within a 45°–53° range. SE, spherical equivalent; AL/R, axial length/radius of curvature ratio; ACD, anterior chamber depth; AQD, aqueous depth; LT, lens thickness; VCD, vitreous chamber depth; CCT, central corneal thickness; RDV-S, superior defocus; RDV-I, inferior defocus; RDV-T, temporal defocus; and RDV-N, nasal defocus.

In the analysis of the relationship between RPR and ocular biometry, RDV45–53 and RDV30–45 showed relatively strong correlations with AL/R, ACD, LT, and VCD (Figure 4). Additionally, LT was significantly correlated with RDV-T and RDV-N in the four quadrants (both *p* < 0.01). In the regression analyses (Table 3), the ocular parameters most closely associated with RDV45–53 and RDV30–45 were AL/R, after adjusting for age and sex (both *p* < 0.01), showing higher correlations than those with RDV15–30, whereas other variables were not significantly associated.

**Table 3:** Multivariable linear regression analysis of ocular biometric parameters with relative peripheral refraction.

Parameter	RDV45–53			RDV30–45			RDV15–30		
	β ± SE^†^	β^‡^	*p*	β ± SE^†^	β^‡^	*p*	*β* ± SE^†^	β^‡^	*p*
Age	0.04 ± 0.03	0.08	0.14	0.03 ± 0.02	0.11	0.06	0.01 ± 0.01	0.07	0.21
Sex	−0.19 ± 0.11	−0.09	0.08	−0.13 ± 0.06	−0.11	0.05	−0.07 ± 0.03	−0.14	0.01*
AL/R	2.82 ± 0.54	0.46	<0.01**	1.60 ± 0.32	0.45	<0.01**	0.41 ± 0.13	0.30	<0.01**
ACD	0.13 ± 0.27	0.32	0.64	0.03 ± 0.16	0.01	0.84	−0.01 ± 0.07	−0.01	0.90
LT	0.57 ± 0.33	0.11	0.08	0.34 ± 0.19	0.11	0.08	0.14 ± 0.08	0.11	0.09
VCD	0.08 ± 0.07	0.10	0.25	0.05 ± 0.04	0.11	0.18	0.03 ± 0.02	0.19	0.04*

*p < 0.05 **p < 0.01; AL/R, axis length/radius of curvature ratio; ACD, anterior chamber depth; LT, lens thickness; VCD, vitreous chamber depth; RDV, refraction difference values. The following ranges were centered on the foveal pit: RDV15–30, within a 15° − 30° range; RDV30–45, within a 30° − 45° range; and RDV45–53, within a 45°–53° range.

All models included age, sex, AL/R, ACD, LT, and VCD.

^†^Unstandardized coefficients β ± SE.

^‡^Standardized coefficients β.

## 4 Discussion

This study confirmed that relative peripheral refraction exhibited hyperopic defocus, which increased with eccentricity from the foveal pit. Children with hyperopia exhibited reduced hyperopic defocus at RDV45–53, whereas differences between emmetropic and myopic children were significant primarily at RDV-T. Among the biometric parameters, AL/R demonstrated the strongest association with peripheral defocus.

The increase in relative hyperopic defocus with eccentricity is consistent with previous MRT-based studies (20, 23, 24) and open-field autorefractor measurements (25, 26) in children. Shen et al. also demonstrated that, as retinal eccentricity increased, image quality declined (27). Wu et al. (23) reported that peripheral hyperopic defocus increased from the center to the periphery and that a statistically significant shift toward hyperopia first occurred at 40° retinal eccentricity using MRT measurements, consistent with our finding that intergroup differences emerged beyond 30° eccentricity. This reinforces the critical role of peripheral optical profiles in refractive development.

Children with hyperopia exhibited reduced relative hyperopic defocus in the peripheral retina compared with myopic or emmetropic peers, particularly beyond 30° eccentricity. This finding is consistent with Chelvin et al. (28), who reported that hyperopic eyes developed a greater peripheral myopia relative to the fovea than emmetropic and myopic eyes, especially at the 45° retinal eccentricity. Although hyperopic defocus predominated across all groups, some hyperopic, emmetropic, and low myopic eyes also exhibited peripheral myopic defocus, consistent with previous findings (29). The predominance of peripheral hyperopic defocus in our hyperopic participants, contrary to the expected peripheral myopic defocus, may reflect a transitional refractive state. Recent MRT-based studies have identified a peripheral shift toward hyperopic defocus before myopia onset (30); therefore, it is plausible that some hyperopic participants were in this transitional phase. Additionally, localized peripheral myopic defocus, even in emmetropic or low myopic eyes, may reflect variations in retinal refraction or asymmetry in peripheral optical profiles.

This reduced peripheral hyperopic defocus in hyperopic eyes may also be partly explained by ocular biometry. Our regression analysis revealed that defocus magnitude in the far peripheral retina (RDV30–45 and RDV45–53) was more strongly correlated with axial elongation, consistent with previous findings (31–33). Compared with axial length alone, the AL/R provides a more comprehensive representation of the refractive structural status of the eye (34). Hyperopic eyes, characterized by shorter axial lengths and smaller AL/R ratios, showed less peripheral hyperopic defocus at large eccentricities, likely due to a relatively flatter posterior pole. For myopic children, those with low myopia showed a significant correlation between SE and RDVN, whereas moderate-to-high myopia had a correlation across multiple retinal regions. This transition also suggests that, as myopia progresses, optical changes may be more significant in wider retinal zones, reflecting progressive eye shape remodeling.

Our study found that SE is associated with TRDV, but recent studies emphasize central retinal defocus feedback as the core mechanism driving myopia development and progression rather than peripheral defocus (8, 35). Persistent hyperopic defocus or blurred images of the central retina, primarily induced by near work or undercorrection or suboptimal correction, disrupts the emmetropization feedback loop governed by homeostasis (36). Within this established paradigm, the observed peripheral hyperopic defocus is viewed as a biomechanical consequence of changes in eye shape, rather than an initiating factor. Therefore, the RPR pattern provides descriptive insights into the eyes with different refractive statuses.

The earliest research on peripheral refraction appeared in Ferree’s work, which described nasal-temporal asymmetry, a pattern that was also identified in our study (37). Nasal hyperopic defocus exceeded temporal hyperopic defocus, and inferior defocus was greater than superior defocus, consistent with the findings of several studies (23, 38). This asymmetry may be attributed to anatomical (39, 40) and optical factors (41). Additionally, relative peripheral hyperopic defocus is more closely associated with horizontal myopia than vertical myopia (40). In our study, the RDV-T positions showed marked differences between the refractive statuses. Studies of adults with myopic anisometropia have shown that RDV-T is closely associated with myopia progression (42), and Xie et al. revealed that RDV-T was the most altered quadrant parameter in the hyperopia–myopia continuum, with the most pronounced changes occurring at the 23-mm axial length threshold, which suggested early involvement in myopia onset (30). This may be due to its role in processing the nasal visual field input, which is the dominant peripheral zone during near work. These findings regarding temporal retinal defocus warrant further investigation to understand its association with refractive development within the context of the central defocus control mechanism.

This study introduced the use of MRT to assess and compare peripheral retinal refraction in children with different refractive statuses, offering a broader assessment of the peripheral refractive state. Nevertheless, this study has several limitations. First, this was a cross-sectional study, and the limited design did not allow for the evaluation of changes over time. Second, although MRT enables efficient wide-field refraction mapping, further research is necessary to obtain quantitative validation against gold-standard techniques across all measured eccentricities. Third, the sample size of the myopic group was approximately three times that of the emmetropic group, which may have reduced the precision of estimates for the latter and could potentially limit the generalizability of our findings to broader populations. Future studies are needed to validate these results with larger and more balanced sample sizes.

In conclusion, peripheral hyperopic defocus increased with eccentricity in children. Hyperopes showed reduced defocus in the far periphery (RDV45–53) compared to those with emmetropia and myopia. AL/R exhibited the strongest correlation with mid-to-far peripheral defocus among the biometric parameters, while temporal defocus (RDV-T) showed the most significant difference among the different refractive statuses. These findings characterize peripheral hyperopic defocus patterns as features that are associated with refractive development.

## Data Availability

The raw data supporting the conclusions of this article will be made available by the authors, without undue reservation.
