# Supplementary material for: Quantitative assessment of relative peripheral refraction in children with different refractive statuses and its associations with ocular biometry
Source: Front Med (Lausanne). 2026 Feb 2;13:1711559. doi: 10.3389/fmed.2026.1711559 (PMC12907144; doi:10.3389/fmed.2026.1711559)
Supplement: Supplementary file 1 [file Table_1.docx]

**Supplement Table 1** Comparison of refraction difference values at different eccentricities and in different quadrants of the eye by myopia subgroups

| Eccentricity  Median (Q1,Q3) | Low Myopia  n=118 | Moderate-to-high Myopia  n=47 | *P*_1_ | *P*_2_ |
| --- | --- | --- | --- | --- |
| RDV15 | 0.14 (0.10,0.17) | 0.15 (0.11,0.20) | 0.37 | 0.54 |
| RDV15-30 | 0.46 (0.36,0.56) | 0.56 (0.37,0.75) | 0.22 | 0.02* |
| RDV30-45^†^ | 1.02 (0.73,1.28) | 1.09 (0.75,1.48) | 0.94 | 0.12 |
| RDV45-53 | 1.22 (0.70,1.76) | 1.23 (0.70,1.88) | 0.70 | 0.38 |
| TRDV^†^ | 0.88 (0.57,1.16) | 0.86 (0.59,1.27) | 0.95 | 0.17 |
| RDV-S | 0.62 (0.38,0.96) | 0.91 (0.51,1.68) | 0.07 | <0.01** |
| RDV-T | 0.30 (0.02,0.61) | 0.36 (0.04,0.74) | 0.41 | 0.10 |
| RDV-I | 0.98 (0.49,1.36) | 0.71 (0.37,1.27) | 0.22 | 0.08 |
| RDV-N | 1.42 (1.09,1.94) | 1.54 (0.84,1.99) | 0.25 | 0.88 |

**P* < 0.05 ***P*<0.01; n, number; AL/R, axial length/radius of curvature; RDV, refraction difference values;

*P*_1_ and *P*_2_ are performed from generalized linear model, adjusted for (sex, age, AL/R) and (sex, age, AL), respectively.

The following ranges were centered on the foveal pit: RDV15−30, within a 15°−30° range; RDV30–45, within a 30°–45° range; RDV45–53, within a 45°–53° range. TRDV, within the total measuring range of a 53° circle. RDV–S, superior defocus; RDV–I, inferior defocus; RDV–T, temporal defocus; RDV–N, nasal defocus.
