# Supplementary material for: Quantitative assessment of relative peripheral refraction in children with different refractive statuses and its associations with ocular biometry
Source: Front Med (Lausanne). 2026 Feb 2;13:1711559. doi: 10.3389/fmed.2026.1711559 (PMC12907144; doi:10.3389/fmed.2026.1711559)
Supplement: Supplementary file 2 [file Table_2.docx]

**Supplement Table 2** Partial correlation analysis between refraction difference values and spherical equivalent under different adjustment models

| Eccentricity | Total Myopia (n=165) | | | | Low Myopia (n=118) | | | | Moderate-to-high Myopia (n=47) | | | |
| --- | --- | --- | --- | --- | --- | --- | --- | --- | --- | --- | --- | --- |
|  | *r*_1_ | *P* | *r*_2_ | *P* | *r*_1_ | *P* | *r*_2_ | *P* | *r*_1_ | *P* | *r*_2_ | *P* |
| RDV15 | -0.15 | 0.06 | -0.08 | 0.31 | -0.06 | 0.55 | 0.03 | 0.78 | -0.26 | 0.09 | -0.25 | 0.10 |
| RDV15-30 | -0.23 | <0.01** | -0.30 | <0.01** | 0.07 | 0.46 | 0.07 | 0.65 | -0.46 | <0.01** | -0.52 | <0.01** |
| RDV30-45 | -0.10 | 0.19 | -0.26 | <0.01** | 0.16 | 0.09 | 0.08 | 0.41 | -0.37 | 0.01* | -0.50 | <0.01** |
| RDV45-53 | -0.04 | 0.65 | -0.17 | 0.04* | 0.18 | 0.06 | 0.13 | 0.17 | -0.30 | 0.04* | -0.48 | <0.01** |
| TRDV | -0.09 | 0.25 | -0.23 | <0.01** | 0.17 | 0.08 | 0.11 | 0.25 | -0.36 | 0.02* | -0.50 | <0.01** |
| RDV-S | -0.25 | <0.01** | -0.45 | <0.01** | 0.12 | 0.22 | -0.09 | 0.37 | -0.53 | <0.01** | -0.52 | <0.01** |
| RDV-T | -0.13 | 0.10 | -0.19 | 0.01* | -0.02 | 0.86 | 0.07 | 0.45 | -0.09 | 0.57 | -0.31 | 0.04* |
| RDV-I | 0.09 | 0.28 | 0.14 | 0.07 | 0.08 | 0.39 | 0.19 | 0.05 | 0.06 | 0.68 | -0.14 | 0.37 |
| RDV-N | 0.02 | 0.82 | -0.12 | 0.12 | 0.24 | <0.01** | 0.11 | 0.26 | -0.32 | 0.04* | -0.39 | <0.01** |

**P* < 0.05 ***P*<0.01; *r*_1_, adjusted for AL/R ratio, age, and sex. *r*_2_, adjusted for AL, age, and sex; n, number; RDV, refraction difference values

The following ranges were centered on the foveal pit: RDV15−30, within a 15°−30° range; RDV30–45, within a 30°–45° range; RDV45–53, within a 45°–53° range. TRDV, within the total measuring range of a 53° circle. RDV–S, superior defocus; RDV–I, inferior defocus; RDV–T, temporal defocus; RDV–N, nasal defocus.
